# Medullary Aplasia Revealing a Metastatic Prostatic Adenocarcinoma

**DOI:** 10.1155/2021/8898130

**Published:** 2021-02-23

**Authors:** Idriss Ziani, Ahmed Ibrahimi, Omar Bellouki, Youssef Zaoui, Yasmine Laraqui Housseini, Fouad Zouidia, Hachem El Sayegh, Lounis Benslimane, Yassine Nouini

**Affiliations:** ^1^Department of Urological Surgery “A”, Ibn Sina University Hospital of Rabat, Rabat, Morocco; ^2^Department of Anatomopathology and Cytology, Ibn Sina University Hospital of Rabat, Rabat, Morocco; ^3^Faculty of Medicine of Rabat, Mohammed V University, Rabat, Morocco

## Abstract

In this case report, we are reporting the case of a 68-year-old male patient who was admitted in our hospital for unintended weight loss, asthenia, and anorexia. Physical examination showed clinical signs of anemia such as pallor of skin and mucous membranes; hemodynamic parameters were normal. Complete blood count (CBC) analysis showed a pancytopenia with anemia, thrombocytopenia, and leukopenia. BM biopsy was performed, showing a malignant infiltration of bone marrow by a metastatic prostate cancer confirmed by immunohistochemistry. Prostate biopsy confirmed the diagnosis of acinar adenocarcinoma with Gleason score 8 (4 + 4), ISUP grade group 4. Our patient underwent chemical castration using LH-RH analogs in association with second-line hormone therapy by abiraterone acetate. The evolution was good on both the oncological and hematological levels.

## 1. Introduction

Prostate cancer is the second most frequently diagnosed cancer in men, with more than 1.2 million new cases worldwide and causing 3.8% of all deaths by cancer in men in 2018 [1].

The highest death rates were recorded in Central America (10.7 per 100,000 inhabitants), followed by Australia and New Zealand (10.2) and Western Europe (10.1) [2]. The 5-year relative survival rate for localized and regional prostate cancer is 100%, compared to 30.5% for metastatic cases [3]. Bones are common metastatic sites and can even be a form of revelation of advanced prostate cancer. [4].

However, metastatic invasion of the bone marrow remains exceptional in prostate adenocarcinoma [5]. This condition can lead to bone marrow failure involving one or more cell lines, which can cause pancytopenia when all the lines are affected. This condition can lead to serious complications and is linked to a poor prognosis [5]. Infiltration of the bone marrow by metastatic tumor cells can lead to bone marrow failure, resulting in hematologic abnormalities. When cancer cells invade healthy bone marrow, they replace hematopoietic stem cells, resulting in the depletion of several cell lines. Nieder et al. describe a typical pattern of anemia, thrombocytopenia, and increased mortality [5].

Although rare, cases of metastatic bone marrow invasion of prostatic adenocarcinoma often present with thrombocytopenia, whether or not associated with anemia [5].

We report a rare case of metastatic adenocarcinoma of the prostate with invasion of the bone marrow discovered in a bone marrow aplasia manifested by pancytopenia, which represents the particularity of our observation. Another peculiarity of our observation is the favorable response to second-line hormone therapy.

To our knowledge, this is the second observation reported in the literature relating to pancytopenia in metastatic prostate cancer.

## 2. Case Report

We report the case of a 68-year-old male patient with history of high blood pressure and hypercholesterolemia, who initially consults for an unintended weight loss estimated at 12 kg in 1 month, asthenia, and anorexia. The patient also reported bone pain in his pelvis.

Physical examination found a patient in poor general condition, with pallor of skin and mucous membranes and normal hemodynamic parameters. A CBC was performed showing white blood cell (WBC) count of 3,500 cells/mL (normal range: 4,000–11,000), platelets of 40,000 cells/mL (normal range: 150,000–450,000), and hemoglobin (Hb) of 5 g/dL (normal range: 13.0–17.0). Faced with this picture, a malignant hemopathy was suspected, after conditioning, of an emergency transfusion with units of red blood cells and platelets, in order to perform a bone marrow biopsy (OMB).

**Figure 1:**
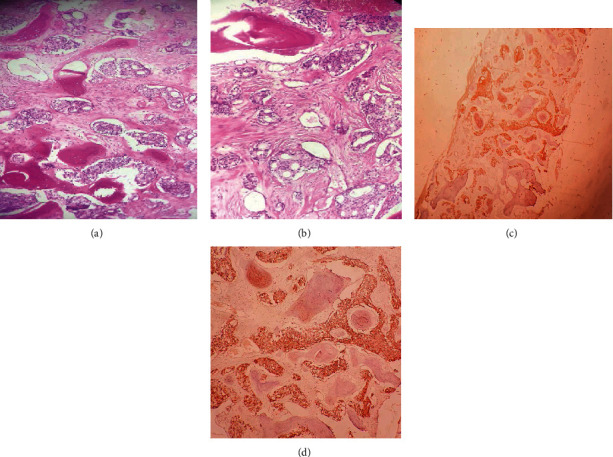
(a, b) Hematoxylin eosin (×100): medullary infiltration by an acinar adenocarcinoma of the prostate. (c) BOM immunohistochemistry: positive labeling of tumor cells by the anti-PSA G antibody (×40). (d) BOM immunohistochemistry: positive labeling of tumor cells by the anti-PSA G antibody (×100).

Pathology study showed malignant infiltration of the bone marrow. The tumor cells are medium sized with a thin cytoplasm. The nuclei are ovoid, finely nucleated, showing many figures of mitosis, these tumor cells probably suspect a prostatic origin, and immunohistochemical staining using prostate-specific antigen (PSA) confirmed the prostatic origin this metastatic invasion (Figure 1).

Rectal examination found hard prostate nodules. The PSA serum concentration was at 197 ng/mL.

**Figure 2:**
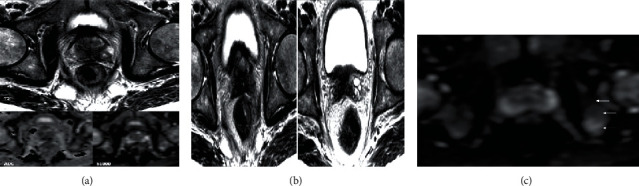
(a) Multiparametric MRI image showing a suspicious nodule with capsular intrusion and invasion of the rectal wall. Nodular image sitting at the level of the left CZ sector of the prostatic base in heterogeneous hyposignal on the T2-weighted images, in hypersignal B1000 diffusion with restriction on the ADC card. Note a rupture of the prostatic capsule with invasion of the left neurovascular strip and thickening of the rectal wall (isosignal T2 and hypersignal in diffusion). (b) T2 sequence of a multiparametric MRI of the prostate objectifying an invasion of the base of the seminal vesicles T2 hyposignal aspect of the base of the seminal vesicles denoting their invasion. (c) B1000 diffusion sequence of a multiparameter MRI objectifying an associated secondary bone involvement, with a heterogeneous aspect of the bone structure.

A multiparametric MRI was performed. On T2-weighted (T2W) images, a PI-RADS V tumor focus in the left basal peripheral zone was detected (Figure 2).

**Figure 3:**
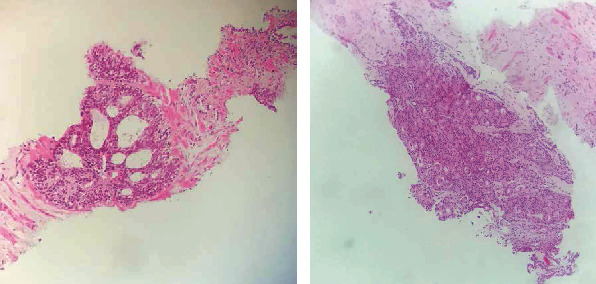
Hematoxylin eosin (×100): infiltration of the prostatic parenchyma by a grade 4 acinar prostatic adenocarcinoma 4.

**Figure 4:**
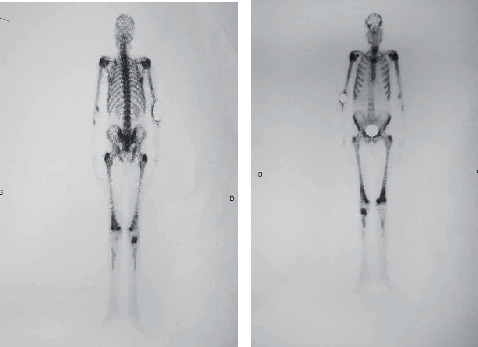
Objective bone scan of many secondary metastatic lesions.

A transrectal ultrasound guided biopsy of the prostate was performed and revealed an acinar adenocarcinoma with Gleason score 8 (4 + 4), ISUP grade group 4 occupying 20% of the biopsy surface on the left, and 10% on the right with perineural neoplastic invasion and invasion of the periprostatic tissue (Figure 3). Bone scan showed several secondary bone locations (Figure 4). Chest-abdomen-pelvis CT did not find any other visceral metastases.

Given the high metastatic volume, the multidisciplinary consultation meeting decided to perform chemical castration using LH-RH analogs in association with second-line hormone therapy by abiraterone acetate after a 15-day antiandrogen treatment to prevent “flare-up.”

Follow-up showed a platelet level of 70,000 cells/mL, and hemoglobin of 11 g/dL with normal WBC three months after treatment. PSA levels drop to 65 ng/mL at 3 months, and 43 ng/mL at 6 months.

## 3. Discussion

Prostate cancer is the second most common cancer in men, and it is the 5th leading cause of death worldwide [5, 16]. Metastatic forms of prostate cancer are linked to a highest rate of morbidity and mortality, and bones remain the most common metastatic site [6].

Depending on the extent of the metastatic lesion and its location in the bone, the consequences on the function of the bone marrow can be variable. Usually, it causes thrombocytopenia and anemia [4, 7]. In the literature, we find that thrombocytopenia and/or anemia are the most common consequences of bone marrow failure due to prostate cancer metastasis. Patients in this condition are reported to have poor prognosis [5, 8].

To our knowledge, pancytopenia due to medullary aplasia from a prostate cancer metastasis has only been reported once in the literature before our paper.

Jacho et al. reported “diffuse invasive carcinoma” as a type of bone metastasis in 1930 [9]. On the basis of this report, Hayashi et al. recommended the term “disseminated carcinomatosis of the bone marrow” (DCBM) with clinical pathological characteristics using the result of the count of diffuse metastases of the bone marrow of solid cancers in Japan in 1979 [9]. Gastric cancer accounts for >90% of all primary lesions, while prostate cancer accounts for only 3.7% [9].

Unfortunately, few data have been published in the literature on the prognosis and survival of the patient with a solid tumor with bone marrow metastases [5, 10].

The mechanism of damage to the bone marrow in case of bone metastases is based on fibrosis, necrosis, and osteoid formation resulting in damage to the hematopoietic function of the peripheral bone marrow.

Bone reactions in metastatic lesions are classified as osteoblastic, osteolytic, intertrabecular, or mixed metastases. Intertrabecular metastasis is a concept similar to DCBM. In the case of intertrabecular metastases, cancer cells are present between the bony trabeculae, and the bone cortex is preserved without destruction. Cancer cells replace bone cells and spread. Intertrabecular metastases are the initial presentation of bone metastases and can then progress to other patterns [5].

However, intertrabecular metastasis leads to DIC when it progresses without change. Older age, man, primary tumor necrosis, and advanced stage have been shown to be risk factors for CAD in solid tumors [9].

Chernow and Wallner recommend performing a bone scintigram and bone marrow biopsy, due to the high possibility of bone marrow metastasis if there are more than two findings among leukoerythroblastosis, platelet count < 100,000/*μ*L, LDH > 500 IU/L, and/or bone pain [9].

Although thrombocytopenia in the context of metastatic disease is most often attributable to an actual invasion of the bone marrow by tumor cells, a review of the literature reveals a small number of cases in which an underlying immune component was suspected. Spivack et al. described 3 cases in which the patients presented both malignant epithelial tumors and a suspected ITP. Of these 3 patients, 1 had an adenocarcinoma of the prostate, and all 3 had isolated thrombocytopenia which responded to treatment with prednisone. Analysis of the bone marrow in the 3 patients showed no signs of replacement by metastatic tumor cells, contrary to the situation of our patient [11].

Contrary to our observation, Geenen et al. have previously shown that the white blood cell system does not appear to be affected in patients with metastatic prostate cancer [5].

Therapeutically, Nieder et al. who retrospectively analyzed 51 patients with anemia and thrombocytopenia by bone metastases, most of the patients (57%) were treated immediately with chemotherapy par by taxotere, and some patients also received second-line treatment with mitoxantrone. Most patients' survival was 2 to 3 years, but 4 patients were alive more than 5 years after detection of bone metastases [4].

Similarly in the 2nd case reported by Kunthur, the patient was treated from the start with a docetaxel chemotherapy with good progress [12].

Our patient was rather treated by a hormone therapy of 2 generations with a good evolution on the biological and oncological plan with a current decline of 12 months.

It should also be noted that androgen-deprivation therapy could lead to a decrease in hemoglobin, for example, an average reduction of 1.1 g/dL in the study by Curtis et al. [13]. Beer et al. observed an average decrease of 0.54 g/dL 3 months after the start of androgen-deprivation therapy [14]. However, the mean level increased in patients with a baseline level < 12 g/dL. A decrease after 3 months was independently associated with shorter survival and progression-free survival. Already in a previous study, the same group had described an association between anemia and shorter survival in men with newly diagnosed metastatic prostate cancer [15].

The delay in correcting anemia in our patient may be partly due to the treatment of hormone therapy.

The peculiarity of our observation, in addition to the fact that it is a rare case, is the response to second-generation hormone therapy, thus delaying chemotherapy.

## 4. Conclusion

Although bone metastases are common in prostate cancer, metastatic involvement of the bone marrow remains relatively rare and reflects an unfavorable course of the disease. The usual translation in the literature is an association between anemia and thrombocytopenia.

Our case has the particularity of reaching the 3 hematopoietic lines which led to aplastic anemia and pan cytopenia, the evolution was pretty good under second-line hormone therapy.
